# Model-Informed Drug Development: In Silico Assessment of Drug Bioperformance following Oral and Percutaneous Administration

**DOI:** 10.3390/ph17020177

**Published:** 2024-01-30

**Authors:** Jelena Djuris, Sandra Cvijic, Ljiljana Djekic

**Affiliations:** Department of Pharmaceutical Technology and Cosmetology, Faculty of Pharmacy, University of Belgrade, Vojvode Stepe 450, 11221 Belgrade, Serbia; gsandra@pharmacy.bg.ac.rs (S.C.); ljiljana.djekic@pharmacy.bg.ac.rs (L.D.)

**Keywords:** model-informed drug development, solubility, drug dissolution, drug release, release mechanism, drug permeation, modeling, oral absorption, percutaneous permeation

## Abstract

The pharmaceutical industry has faced significant changes in recent years, primarily influenced by regulatory standards, market competition, and the need to accelerate drug development. Model-informed drug development (MIDD) leverages quantitative computational models to facilitate decision-making processes. This approach sheds light on the complex interplay between the influence of a drug’s performance and the resulting clinical outcomes. This comprehensive review aims to explain the mechanisms that control the dissolution and/or release of drugs and their subsequent permeation through biological membranes. Furthermore, the importance of simulating these processes through a variety of in silico models is emphasized. Advanced compartmental absorption models provide an analytical framework to understand the kinetics of transit, dissolution, and absorption associated with orally administered drugs. In contrast, for topical and transdermal drug delivery systems, the prediction of drug permeation is predominantly based on quantitative structure–permeation relationships and molecular dynamics simulations. This review describes a variety of modeling strategies, ranging from mechanistic to empirical equations, and highlights the growing importance of state-of-the-art tools such as artificial intelligence, as well as advanced imaging and spectroscopic techniques.

## 1. Introduction

Formulation development, one of the most challenging activities in the pharmaceutical industry, has changed significantly over the years. This situation is a consequence of continuous pressure from regulators, increasing competition between companies, and the constant struggle to speed up formulation development and launch a drug product in a shorter time frame. Among a number of innovations that have been introduced in the pharmaceutical industry in recent years, in silico computational modeling deserves special attention. In silico modeling in pharmaceutical development, also known as model-informed drug development (MIDD), is a relatively broad concept, defined as the “application of a wide range of quantitative models in drug development to facilitate the decision-making process” [1] or “development and application of exposure-based, biological, and statistical models derived from preclinical and clinical data sources” [2].

In general, MIDD methods refer to the application of various computational tools to mathematically describe the relationship between different factors (drug, formulation, organism, and/or disease-related) that influence the bio-performance of a drug and the associated clinical outcomes, with the ultimate goal of supporting optimal decision-making during formulation development, thus increasing the chance of a product receiving approval and recouping the investment in its development.

MIDD tools are often available as ready-to-use software, although the use of in-house designed (generic) models is also a common practice [3,4,5,6]. Moreover, some models are mechanistic in nature, i.e., they allow interpretation of the underlying processes that influence system response, but some operate as ‘black boxes’ where the algorithms in the background are not revealed to users. In any case, the user needs to understand the basic scientific principles and, in the case of mechanistic models, the mathematical equations incorporated in an in silico model.

This review focuses on elucidating the key processes that control drug release and permeation through biological membranes following oral and cutaneous application and discusses the possibilities of simulating these processes using available in silico tools. Selected examples are also provided to illustrate current trends in MIDD practice.

## 2. Interpretation of Oral Drug Dissolution, Permeation, and Absorption within Physiologically-Based Biopharmaceutics Modeling

Physiologically-based biopharmaceutics modeling (PBBM) is an emerging MIDD tool used for mechanistic interpretation and prediction of drug absorption, distribution, metabolism, and excretion (ADME), with a particular focus on establishing a link between bio-predictive in vitro dissolution testing and mechanistic modeling of drug absorption [7]. PBBM is widely used by pharmaceutical companies, research institutions, and medical regulatory authorities and is now an indispensable tool at various stages of drug and formulation development [8,9,10,11,12]. A major advantage of PBBM over conventional in vitro and preclinical animal studies is the ability to link the physicochemical properties of a drug to its dissolution, absorption, and disposition in a target patient or population, taking into account specific physiological conditions. This is achieved through linked differential equations that describe simultaneous or sequential dynamic processes that a drug undergoes in the body. In addition, PBBM predictions can relate to different physiological or disease states, so this unique approach can support personalized pharmacotherapy and drug/dose/dosing regimen selection in different patient populations or individual patients [13,14,15].

**Figure 1:**
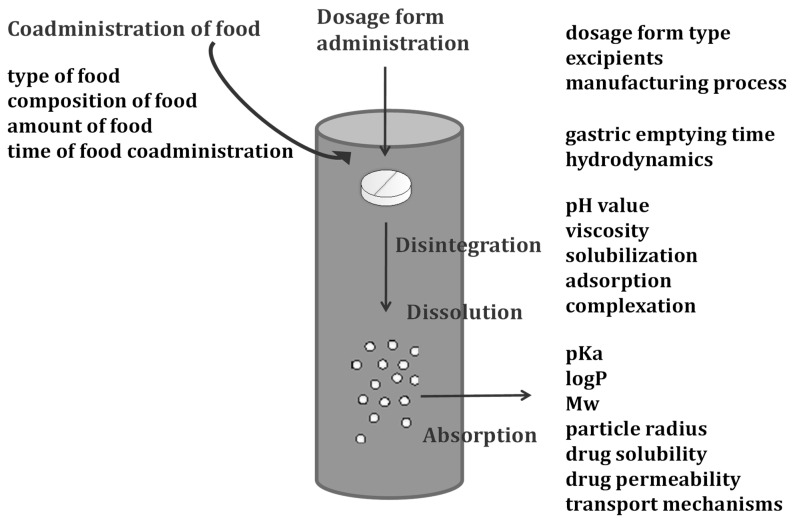
Factors and processes affecting drug/dosage form performance in the GI tract following peroral administration.

Although PBB models can represent different routes of drug administration, they have mainly been used to simulate the bio-performance of drugs following peroral administration [3,5,11,16]. In this case, the processes that a drug undergoes in the body are influenced by a number of factors that reflect the drug properties, critical product attributes, and features of human gastrointestinal (GI) tract physiology (Figure 1). These factors are treated as input parameters in PBBM, i.e., each model is drug-specific and is further customized for a particular formulation type and physiology or disease state. To simulate the overall influence of these parameters on drug absorption, a PBB model must mathematically describe each process occurring in the GI lumen, either simultaneously or concurrently.

In order for a drug substance to be absorbed, it must first be released from a dosage form and dissolved in the body fluids, then, diffuse to the site of absorption and, finally, pass through the biological membrane and enter the enterocytes. Depending on the dosage form, a drug may be readily available for dissolution (e.g., immediate release (IR) powders), or a dosage form needs to disintegrate and release drug particles prior to dissolution. The drug release rate can be modeled using various functions that will be described in the following sections, and they can be included in a PBB model. In addition, a systematic review of disintegration mechanisms and mathematical models describing these mechanisms is provided by Markl and Zeitler [17]. There are also specialized software tools, such as DDDPlus^TM^ software, which considers more specific formulation parameters, including types and amounts of excipients and manufacturing properties (e.g., compression force, tablet diameter) to simulate tablet disintegration and drug particle release rate [18]. In particular, DDDPlus^TM^ software considers that the main mechanism of tablet disintegration is the swelling of the disintegrant particles caused by water uptake into the tablet, which generates a force inside the tablet that eventually leads to the breakage of the bonds between the particles [19].

Dosage form disintegration enables drug dissolution, which is one of the key factors governing drug systemic exposure. Although the term ‘drug dissolution’ is sometimes confused with ‘drug release’, these terms have different meanings, with ‘drug release’ being a more complex phenomenon involving the ‘dissolution of drug particles’ [20].

**Figure 2:**
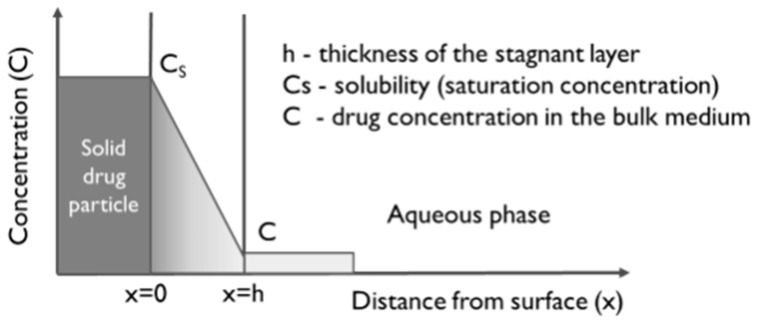
Representation of mass transfer through stagnant diffusion layer.

Different equations have been used to describe the dissolution of drugs in PBBM, and most of them are based on the mass transfer model (Table 1). In this model, mass transfer is driven by a concentration gradient, i.e., a saturated solution forms at the particle surface, and the dissolution of the drug is controlled by the diffusion of molecules through the stationary liquid layer surrounding each particle (Figure 2).

As already mentioned, drug dissolution is a prerequisite for absorption but acts as a rate-limiting factor for absorption when this process is slower than permeation. In such cases, all factors affecting drug dissolution (including drug, formulation, and physiological factors) need to be carefully evaluated and considered in a PBB model.

Solubility is a critical parameter affecting drug dissolution, meaning that for poorly soluble drugs, solubility may limit dissolution. In addition, the solubility of weak electrolytes changes with the pH of the medium. Since the pH of gastrointestinal fluids varies greatly, it is important to study the solubility of drugs under different pH conditions in the physiological range.

A common formulation approach to increase the absorption and bioavailability of drugs, especially for poorly soluble drugs, is to use salt forms, which generally have better solubility than the acidic or basic drug form. However, a salt form may precipitate in the GI tract, e.g., if its solubility in the stomach is much higher than in the small intestine. This highlights the importance of considering the precipitation rate of drugs in bio-predictive dissolution models. It should also be noted that the drug precipitation rate depends on the presence of excipients and the regional conditions (pH value, volume, etc.) in the GI tract. Various methods have been proposed to address these issues, including both in vitro assays [22,23] and mathematical predictions [24,25,26]. Consequently, the precipitation process has been considered in the dissolution models integrated within PBBM software [27,28,29,30].

Solubility may also vary between different polymorphic forms of a drug, and similar considerations apply to hydrates (which generally have lower solubility and dissolution rate in aqueous solutions) and the amorphous state (which is characterized by increased solubility and dissolution rate compared to the drug’s crystalline forms). However, some drug forms may undergo transformation in the GI lumen. For example, anhydrous carbamazepine tends to convert to a hydrate form upon contact with GI fluids, and this may compromise the drug dissolution rate [31]. Such phenomena can be accounted for in a PBB dissolution model by entering different solubility values for different drug forms.

Another phenomenon that can either increase or decrease the solubility of drugs is complexation with excipients or other compounds present in the GI tract. A typical example of the positive effect on drug solubility is the complexation of lipophilic drugs with cyclodextrins (CDs). The effect of CD on drug solubility can be estimated from the drug solubility in the absence of CD and the binding constant for a given system [32,33,34]. For example, if the stoichiometry of the drug–CD complex (D/CD) is 1:1, the total drug solubility (*C_Dtot_*) can be calculated using the following equation [34]:

(1) $$C_{Dtot}=C_{D}+\frac{K\times C_{D}\times C_{CDtot}}{1+k\times C_{D}}$$

where *K* is equilibrium binding constant, *C_D_*—concentration of free dissolved drug (drug intrinsic solubility), and *C_CDtot_*—total CD concentration. The binding constant can be estimated from the slope of the phase solubility (solubility vs. CD concentration) diagram, using Equation (2) [32,33,34]:

(2) $$K=\frac{Slope}{C_{D}\times \left(1+Slope\right)}$$

Moreover, molecular docking can serve as a useful in silico technique for predicting interactions between CDs and lipophilic drugs, elucidating the molecular mechanisms of drug encapsulation within CDs of varying sizes (α, β, γ). This technique can be utilized to generate and cluster poses based on free energies, subsequently analyzing representative poses to understand the binding affinities and interactions between drugs and CDs. Through docking, we can assess CDs’ impact on guest molecule properties such as solubility, stability, and other physical and chemical characteristics [35]. Such simulations can also highlight the cavity size-dependent stoichiometry of drug−CD complexation, offering a pictorial representation of the diverse interactions between drugs and α-, β-, and γ-CDs [36].

The presence of surfactants, either in the formulations or in the GI tract (e.g., naturally occurring bile salts), may also affect drug solubility and dissolution. These surface-active agents increase drug solubility and dissolution by facilitating particle wetting (thus increasing the effective surface area in contact with the solvent) or by micellar solubilization, depending on the surfactant concentration. Drug solubility in the presence of bile salts (*C_s_*_(*BS*)_) can be estimated using Equation (3) [37]:

(3) $$C_{s\left(BS\right)}=C_{s}+SC_{aq}\times SR\times M_{w}\times \left[BS\right]$$

where *C_s_* is drug solubility in the absence of bile salts, *SC_aq_*—aqueous solubilization capacity for the drug, *SR*—bile salts solubilization ratio for the drug, *M_w_*—drug molecular weight, and [*BS*]—concentration of bile salts.

Here, *SC_aq_* is expressed as the ratio of moles of drug and moles of water at drug aqueous solubility concentration:

(4) $$SC_{aq}=\frac{moles\left(drug\right)}{moles\left(water\right)}$$

and *SR* can be calculated from the octanol/water partition coefficient for the drug (P) using Equation (5):

(5) logSR=2.23+0.61×logP

On the other hand, a drug trapped in a micelle diffuses slower in comparison to the free drug, as illustrated in Equation (6) [38].

(6) $$D_{eff}=D\times f+D_{mic}\times \left(1-f\right)$$

where *D_eff_* is effective diffusion coefficient, *D*—diffusion coefficient of free drug, *D_mic_*—diffusion coefficient of drug associated with bile salt micelles, and *f*—fraction of free drug, which is expressed as:

(7) $$f=\frac{C_{s}}{C_{s\left(BS\right)}}$$

Still, an increase in solubility prevails, so the overall effect of bile salts on poorly soluble drugs is an increased dissolution rate [38].

Particle size is another key factor that affects drug dissolution. Smaller particles have a larger surface area that comes into contact with the solvent, so reducing the particle size usually increases the drug dissolution. This is one of the most important formulation strategies to increase the absorption of poorly soluble drugs, especially in the case of high-dose oral dosage forms. However, if the particles are too small and hydrophobic in nature, they may form aggregates, leading to a decrease in the effective surface area and, consequently, dissolution. Therefore, the effect of decreased particle size should be carefully considered in the early phase of formulation development. In this context, PBBM is particularly useful as it allows an estimation of the impact of particle size on drug dissolution and absorption prior to formulation changes. This type of sensitivity analysis (SA) can be performed by gradually changing the drug particle size while keeping the other input parameters at baseline values and assessing the impact on the extent and rate of drug absorption. Several case studies from the industry demonstrate the successful application of PBBM SA for the selection of drug particle size and the establishment of drug particle size specifications [39,40,41].

Drug dissolution can also be affected by the particle shape. This effect is often neglected in dissolution models, which generally assume a spherical particle shape. However, there have been attempts to include the shape factor in the dissolution equations [42,43,44]. There are also reports on more complicated computational methods to estimate the dissolution of particles with irregular shapes, such as the computational fluid dynamics-direct numerical simulation method (CFD-DNS) [45] or the moving particle semi-implicit (MPS) method [46]. In addition, the particle shape can change over time. This phenomenon is more difficult to understand and incorporate into a dissolution model, but there are a few examples of dissolution modeling that consider particle shape changes over time [43,47].

There are some situations where the dissolution model has to take into account more specific phenomena, e.g., in the case of nano-sized drug particles. Indeed, it has been shown that nanoparticles have a much higher gastrointestinal tissue uptake and bioavailability compared to micro-sized particles [48,49]. This phenomenon cannot be explained solely by the increase in surface area due to reduced particle size, supporting the hypothesis that nanoparticles may form a supersaturated solution in a small volume of fluid between the intestinal microvilli, resulting in increased drug flux across the apical membrane. In such cases, empirical equations may be derived and included in a PBB model to estimate nanoparticles’ effect on drug dissolution and concomitant absorption. Such an approach is illustrated in the study by Zhang et al. [50] using a poorly soluble, poorly permeable, and weakly basic drug. These authors utilized PBBM to demonstrate how particle size reduction to the nano-sized range can be a beneficial strategy to increase the bioavailability of a poorly soluble, poorly permeable, weakly basic drug with pH-dependent solubility in the physiological range.

## 3. Drug Release Modeling

Drug release modeling is of pivotal importance for understanding the release mechanism and predicting the release of drugs from different formulations, carriers, and dosage forms. Modeling of drug release is particularly important for modified release dosage forms specifically developed to provide prolonged, controlled, sustained, delayed, pulsatile release or other types of release profile modifications. In conventional dosage forms that are administered orally, there are two specific types of delivery systems: monolithic and multiparticulate [51]. Matrix tablets are monolithic forms that can be formulated with a variety of polymers (hydrophilic and hydrophobic) or lipid excipients. Multiparticulate systems can be tailored to different release profiles, depending on the manufacturing technology and the specific properties of the drug and product being developed. Pellets are the most commonly used multiparticulate system for modified drug release.

The ability to accurately model drug release kinetics enables the optimization of drug delivery systems, ensuring efficacy and minimizing adverse effects. Over the years, various methods have been developed to address this critical aspect of pharmaceutical research. They range from mechanistic and empirical approaches to hybrid models that combine the strengths of both approaches. In addition, the emergence of artificial intelligence (AI) and machine learning (ML) techniques has led to the development of innovative solutions for drug release modeling.

Traditionally, the following mechanisms have been studied and predominantly utilized to control drug release: diffusion, dissolution, swelling, erosion, osmosis, partitioning, and chemical reactions [52]. In addition, changes in the dosage form/delivery system geometry, phase transition(s), microenvironment pH, and/or ionic strength change or other phenomena can also influence drug release. Mechanistic drug release modeling involves the application of fundamental principles and physicochemical processes to describe the release of drugs from pharmaceutical formulations. At its core, this approach aims to understand the underlying mechanisms that control drug dissolution and diffusion, as well as the interaction between the drug and its delivery system.

Commonly used mechanistic models are based on Fick’s law of diffusion, describing the process of the drug flux *J* as the rate of transfer (*dQ*/*dt*) through the unit surface *A*:

(8) $$J=\frac{dQ}{Adt}=-D\frac{\partial C}{\partial x}$$

where *D* is the diffusion coefficient, and $\frac{\partial C}{\partial x}$ is the change of concentration *C* in the direction *x*. The minus sign represents diffusion gradient, i.e., decrease in the concentration from the higher to the lower.

Since the diffusion of the drug occurs in all three dimensions, the following form of partial equation describes the system more accurately:

(9) $$\frac{\partial C}{\partial t}=D\frac{\partial^{2}C}{\partial x^{2}}+D\frac{\partial^{2}C}{\partial y^{2}}+D\frac{\partial^{2}C}{\partial z^{2}}$$

In order to solve the partial differential equations, initial and boundary conditions need to be considered. In the case of sphere or cylinder geometry of the dosage form (or delivery device), the following equations apply:

(10) $$\frac{\partial C}{\partial t}=D\left(\frac{\partial^{2}C}{\partial r^{2}}+\frac{2}{r}\frac{\partial C}{\partial r}\right)\;\;\;\;\;\;\;\;\;\;\;\left(for\;sphere\;geometry\right)$$

(11) $$\frac{\partial C}{\partial t}=D\left(\frac{\partial^{2}C}{\partial r^{2}}+\frac{1}{r}\frac{\partial C}{\partial r}\right)\;\;\;\;\;\;\;\;\;\;\left(for\;cylinder\;geometry\right)$$

When a constant diffusion coefficient *D* is considered, solutions are derived for a variety of geometries with different initial and boundary conditions. The boundary conditions can be considered as (in)finite as well as (non)sink. Partial differential equations can be solved analytically or numerically with explicit or implicit solutions.

Siepmann and Siepmann [53] provided an overview of models for drug diffusion from controlled drug delivery systems of the matrix or reservoir type. Depending on whether the drug concentration ($c_{ini}$) in the delivery system exceeds the saturation solubility ($c_{s}$) or not, reservoir devices can be considered with non-constant activity sources ($c_{ini}$ < $c_{s}$) or with constant activity sources ($c_{ini}$ > $c_{s}$). Similarly, matrix systems can be considered as monolithic solutions ($c_{ini}$ < $c_{s}$) or monolithic dispersions ($c_{ini}$ > $c_{s}$).

Table 2 provides an overview of the diffusion models for reservoirs or matrix systems with different geometries: slab (thin film), sphere, or cylinder. Tablets usually have the shape of a cylinder, pellets and other multiparticulate systems are considered spheres, while films or semisolid formulations applied in a thin layer (e.g., creams, ointments) can be considered slabs.

In the case of reservoir devices with non-constant activity source ($c_{ini}$ < $c_{s}$), the model actually represents a process of first-order kinetics:

(12) $$\frac{dM_{t}}{dt}=\frac{ADK\left(M_{0}-M_{t}\right)}{VL}$$

whereas, in the case of reservoir devices with constant activity source ($c_{ini}$ > $c_{s}$), the model represents a zero-order kinetic process:

(13) $$\frac{dM_{t}}{dt}=\frac{ADKc_{s}}{L}$$

Similarly, slab-geometry of monolith dispersions matrix system is a type of system where the diffusion model has been described by the Higuchi equation:

(14) $$M_{t}=A\sqrt{Dc_{s}\left(2c_{ini}-c_{s}\right)t}$$

The Higuchi equation was derived for an ointment with a suspended drug.

There are reports of more detailed approaches to solving drug release models based on specific delivery systems, available data, and preconceived assumptions. For example, Jain et al. [54] developed a model that can discriminate between the effects of diffusion and dissolution of the drug encapsulated by the porous passive layer of an implant. The authors found that the dimensionless initial concentration plays a key role in determining whether the problem is diffusion- or dissolution-limited. Chakravarty and Dalal [55] developed a two-phase model for drug release from microparticles with combined effects of solubilization and recrystallization.

One of the main advantages of mechanistic modeling is that it provides valuable insights into the factors that influence drug release and, thus, supports the development and optimization of drug delivery systems. It also enables the prediction of release profiles under different conditions. However, mechanistic modeling can be challenging due to the complex interplay of factors involved, often requiring extensive experimental data for accurate parameter estimation. Furthermore, relying on simplifying assumptions may lead to discrepancies between model predictions and real-world observations. Nonetheless, the rigorous foundation of mechanistic drug release modeling remains a fundamental tool in the development of efficient and reliable drug delivery systems. 

Some of the assumptions made in the previously described diffusion models include considerations of the constant diffusion coefficient [56], one-dimensional diffusion, neglecting of excipients dissolution/diffusion, etc. Different forms of changes in the diffusion coefficient can be derived, depending if the carrier is (micro)porous, non-porous, or highly swollen.

The Siepmann–Peppas sequential layer model [57] enables the understanding and prediction of drug release from hydrophilic matrix tablets subject to swelling and erosion. This model considers diffusion of water and drug with time- and space-dependent diffusion coefficients, moving boundary conditions, swelling of the system, dissolution of both the polymer and the drug, and radial and axial mass transport (demonstrated in the case of cylindrical matrix tablets). Due to the complexity of such a model, Fick´s law is usually derived into simpler semi-empirical forms, which are often used to fit the experimental data due to their simplicity. However, care must be taken in the application and interpretation of such models to take into account the assumptions made in simplification. As mentioned earlier, geometry is critical to the meaningful modeling of the drug release from a delivery system. Delivery systems may vary in complexity of composition. They range from inert structures to systems that undergo changes in their volume, size, and/or shape. These changes are primarily influenced by swelling and erosion processes. Here, models should be used that can capture the diffusivities of the drug and carrier (e.g., polymer) that occur simultaneously.

Empirical drug release modeling methods rely on experimental data to establish mathematical relationships between the drug release profiles and various factors affecting the release process. In contrast to mechanistic models, which focus on the underlying physical and chemical principles, empirical approaches are data-driven and do not require detailed knowledge of the release mechanism. These models are particularly useful when the underlying release mechanism is complex or not well understood, making it challenging to develop a mechanistic model. Empirical modeling often involves curve-fitting techniques, such as polynomial equations, exponential functions, or power laws, to the experimental data to accurately capture the release behavior.

The famous Peppas equation is an example of a semi-empirical model describing the release rate as a total quantity of the drug released being proportional to the power of time, where the power is dependent on the geometry of the formulation and is also indicative of the release mechanism:

(15) $$M_{t}=kt^{n}$$

where $M_{t}$ is the amount of drug released at time *t*, k is a constant, and n is a release-indicating exponent.

Interpretation of the release mechanism type based on the value of the release-indicating exponent *n* depends on the delivery system geometry (Table 3).

For anomalous transport, i.e., combined mechanisms of the drug release, it may be beneficial to utilize the Peppas–Sahlin equation where the release exponent m is used in combination with constants $K_{1}$ and $K_{2}$ to describe the relative contributions of the drug released by diffusion through the (swollen) layer and the erosion, i.e., relaxation, usually of the polymer chains:

(16) $$M_{t}=K_{1}t^{m}+K_{2}t^{2m}$$

In the case of *Case II* transport, constant drug release is achieved, and the equation effectively becomes a zero-order kinetics model. Similarly, in the case of thin film geometry, where the release mechanism is pure diffusion, the equation corresponds to the Higuchi model. It is usually recommended to consider the first 60% of the active ingredient release profile for modeling the mechanism(s).

There are other, more complex forms of semi-empirical models, e.g., for matrix systems where the porosity and tortuosity of the matrix are also considered or for systems with a constant release surface. Similarly, lag time or burst release may also be included in the model equations. Geraili and Mequanint [58] demonstrated that models such as Hixson–Crowell and Hopfenberg can successfully capture polymer erosion as the predominant release mechanism from the photo-crosslinked polyanhydride matrix tablets.

A well-known purely empirical model is the Weibull equation, which can be used to describe different drug release profiles but does not provide insight into the actual release mechanism:

(17) $$M_{t}=1-exp\left[\frac{-{\left(t-T_{i}\right)}^{b}}{a}\right]$$

where $T_{i}$ represents the lag time (typically 0), a describes the time scale of the process, and b is representative of the distribution type (b = 1 in the case of exponential distribution, b > 1 in the case of sigmoid, and b < 1 in the case of parabolic curve).

In addition to the release mechanisms described above, a variety of drug delivery systems have been developed in which a specific stimulus triggers the drug release, such as a change in pH, temperature, or other similar factors [59]. In addition to internal stimuli for drug release, external stimuli, such as (electro)magnetic field, ultrasound, light, etc., can also trigger drug release [60,61,62]. Drug release from complex delivery systems is sometimes based on several processes, including the combination of permeation and diffusion from emulsion-based systems, or is tailored to follow a circadian or other rhythm [63]. 

Sirousazar [64] developed a spherical, temperature-responsive drug delivery system that has an inner layer that undergoes a solid–liquid phase transition at a certain temperature. The response of the system to the temperature change was mathematically modeled by solving the relevant heat and mass transfer equations in a pseudo-steady state. Kashkooli et al. [60] gave an overview and several examples of modeling targeted-release drug delivery systems.

### 3.1. Other Contemporary Approaches to Drug Release Modeling

Computational fluid dynamics (CFD) is a numerical technique for simulating and analyzing fluid flow phenomena. In the context of drug release modeling, CFD enables the study of the interaction between the drug released from the carrier (the dosage form) and the surrounding medium (the fluid) by understanding the flow patterns and dynamics in and around the drug delivery systems. CFD can provide insight into the different mass transport mechanisms responsible for drug release, such as diffusion, convection, and erosion, as well as simulate how different hydrodynamic factors might impact drug release. Other methods used for numerical simulations are based on the discrete element method (DEM), finite element method (FEM), etc.

Kubinski et al. [65] utilized a CFD approach to model the mass transfer coefficients and corresponding drug release for the USP apparatuses I and II configurations of interest. Limited experimental dissolution data was necessary to achieve high predictability for erosion-based formulations. Lou and Hageman [66] investigated the influence of tablet position in the USP apparatus II vessel on polymer erosion and drug release of a surface erodible sustained-release tablet using CFD. The authors developed a mathematical model to describe the polymer erosion and tablet deformation based on the mass transfer coefficient. Numerical analysis was used to simulate the drug release, considering both drug diffusion and polymer erosion as controlling factors. The results showed that tablets located at the off-center position deformed faster than those located at the center position. However, the tablet location had no profound impact on the drug release rate since all drug release profiles were similar. Walsh et al. [67] performed a computational study on paracetamol diffusion from a porous matrix. The authors developed a numerical CFD model based on the finite element method in order to solve the mass transfer equations. Schütt et al. [68] used CFD to evaluate the drug dissolution/tablet disintegration process under the influence of hydrodynamic and shear stress, i.e., the combination of motility patterns and fluid viscosity, aiming to mimic the ascending colonic environment. The motility patterns used were derived from in vivo data, representing different motility patterns in the human ascending colon. They showed significant differences in the drug release rate from the tablets, as well as in the ability of the drug to distribute along the colon.

CFD has also been utilized to simulate hydrodynamic conditions in different dissolution testing apparatuses [69,70,71].

Kalný et al. [72] combined two approaches in a combined simulation of tablet disintegration and ibuprofen dissolution. Tablet fragmentation triggered by swelling of the disintegrant (croscarmellose sodium) was simulated using the discrete element method (DEM), while the dissolution of ibuprofen from the resulting population of disintegrated fragments was simulated using a finite volume grid-based model. The final ibuprofen release curve was then determined by superimposing the dissolution curves of the individual disintegration fragments. This approach facilitates the development of immediate-release solid dosage forms. Kimber et al. [73] used DEM to generate an unstructured mesh over which mass transfer equations were solved to model swelling and drug release from tablets.

Ranjan and Jha [74] used FEM simulations and experiments to study drug release from controlled-release polymeric formulations in a rotating paddle apparatus. The interaction between the hydrodynamics inside the vessel and the swelling and erosion of dosage forms often leads to significant deviations from the dissolution behavior observed when using the approximation of a perfect sink. The authors investigated in detail the effects of agitation speed, drug loading, and polymer swelling and erosion on drug release.

Advances in instrumental techniques, such as spectroscopy, have provided new insights into the study of dissolution phenomena. Van Haaren et al. [75] reviewed ATR-FTIR (attenuated total reflectance-Fourier transformed infrared) spectroscopic imaging in studying underlying chemical and physical mechanisms of drug release from solid dosage forms. Spectroscopic ATR-FTIR imaging can characterize a sample with high chemical specificity and high spatial resolution, and generally, a flow cell set-up is used for ATR-FTIR imaging during dissolution. In addition, a UV-Vis spectrophotometer can be integrated into the pipeline, allowing simultaneous measurement of total drug release during the dissolution process [75]. For ATR-FTIR imaging, the analyzed samples must be placed on an internal reflectance element, and measurements are usually taken at the interface between the analyzed sample and the medium. This method has been used to investigate the influence of pH and/or ionic strength on drug release from a hydrophilic polymer matrix system [76,77], to study the drug release from multi-layer and/or multi-drug tablets [78,79], as well as thin films [80]. In addition, ATR-FTIR imaging was coupled with DEM to model the release of nicotinamide from HPMC matrix tablets [81]. In addition to FTIR spectroscopic imaging, magnetic resonance imaging (MRI), nuclear magnetic resonance (NMR), Raman imaging, coherent anti-Stokes Raman scattering microscopy, UV imaging, fluorescence imaging, terahertz (THz) imaging, and X-ray micro-tomography, are also used to study the dissolution process [75,82]. Brown et al. [82] reviewed various applications of UV(-Vis) dissolution imaging for pharmaceutical systems. Table 4 shows selected examples of imaging studies used to analyze the dissolution process.

#### 3.1.1. Artificial Intelligence and Machine Learning Algorithms in Drug Release Modeling

Specific AI techniques used in drug release modeling include a wide range of algorithms, including neural networks, support vector machines, random forests, and genetic algorithms. These techniques are able to handle complex and nonlinear relationships between different formulation parameters and drug release profiles, leading to improved modeling results. The benefits of using AI in drug release modeling include the ability to process large and diverse datasets, recognize patterns, and make accurate predictions, even in the absence of explicit mechanistic knowledge. AI models can efficiently learn from complex data and offer a data-driven approach that complements traditional mechanistic and empirical modeling techniques. However, challenges arise in the interpretability of AI models, as their internal workings may not be easily understood by researchers or regulatory authorities. Jiang et al. [91] reviewed emerging AI technologies used in solid dosage form development, including their use for predicting drug release profiles. Several ML algorithms used for drug release prediction are presented in the review, including ANNs, ensemble of regression trees, support vector machines, dynamic neural networks, decision trees, self-organizing maps, and deep neural networks. Similarly, Wang et al. [92] provided a state-of-the-art review of ANNs used for the prediction, characterization, and optimization of pharmaceutical formulations. Additional reviews and illustrative examples of a variety of ML algorithms that have been used to model the release profiles of drugs (or other bioactive compounds), including artificial neural networks, decision trees or random forests, adaptive neuro-fuzzy inference systems (ANFIS), genetic programming, etc. can be found elsewhere [93,94,95,96,97].

Some interesting recent examples include a study in which random forest was applied to classify dissolution profiles into two categories: “spring-and-parachute” and “maintenance supersaturation” for the dissolution behavior of solid dispersions [98]. In addition, authors have used random forest as a regression model for the successful prediction of drug release profiles. Elbadawi et al. [99] developed ML models for predicting drug dissolution of 3D printed tablets based on measurements of the viscosity of the extrudable formulations prepared for the fused deposition modeling. Nagy et al. [100] used partial least squares regression and an ANN algorithm to successfully model the dissolution of tablets using NIR and Raman spectra of intact tablets. Recently, Greenberg et al. [101] demonstrated the potential of AI to support precise drug delivery from extracellular vesicles.

In the context of drug release prediction, ANNs are usually considered “black-box” models, where the equations behind the predictions are not visible to the user of the model, nor are they based on the principle of a release mechanism. To assess the interpretability and reliability of explainable AI models, researchers can compare the explanations provided by the AI model with existing mechanistic knowledge. The consistency between the knowledge generated by the AI and established scientific principles confirms the reliability of the model’s predictions. In addition, the accuracy and consistency of the AI model’s predictions across different datasets and experimental conditions can further reinforce the model’s reliability. Explainable AI has found valuable use cases in the optimization of oral drug delivery systems. By analyzing the model’s explanations, researchers can identify key formulation parameters that affect drug release, such as excipient composition, particle size, and matrix properties. This information can guide formulation development and enable the development of oral drug delivery systems with desired release profiles, improved bioavailability, and reduced side effects. Moreover, explainable AI can help explore the effects of different excipients and dosage forms, leading to the discovery of novel drug delivery strategies that maximize therapeutic efficacy. Overall, AI and explainable AI approaches are very promising in drug release modeling. They are advancing pharmaceutical research and ushering in a new era of precision drug delivery.

#### 3.1.2. Hybrid Drug Release Models

**Figure 3:**
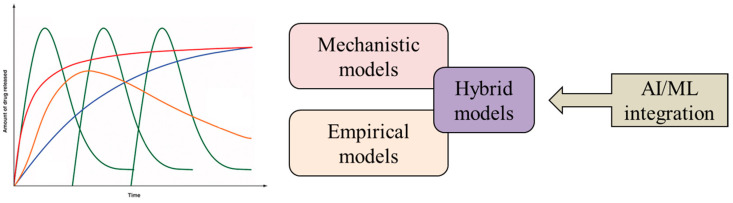
Drug release modeling approaches.

A hybrid approach that combines the strengths of both empirical and mechanistic modeling can be a promising solution. It provides a balance between simplicity and mechanistic understanding, improving the accuracy and predictive ability of drug release models. In hybrid models, mechanistic principles are integrated with empirical data, allowing a more accurate representation of the complex interactions that determine the release of drugs from pharmaceutical formulations. By incorporating mechanistic aspects, such as diffusion as a release mechanism, the model can capture the underlying physical and chemical processes involved in drug release. At the same time, empirical elements are used to fine-tune the model based on experimental data, taking into account specific formulation features and other variables that may not be fully captured by the mechanistic components alone. Different approaches to drug release modeling are represented in Figure 3.

An example of a hybrid model was presented by Yokoyama et al. [102]. The authors investigated bi- and triple-layer tablet formulations containing acetylsalicylic acid and mefenamic acid using cellular automata, a computational model for discrete dynamic systems. This approach allowed them to estimate the effects of the inert layer between the different active ingredients on the release of each active ingredient. In addition, models based on the Noyes–Whitney equation in combination with a cellular automata-based numerical solution for dissolution and disintegration were also developed. Sivasankara and Jonnalagadda [103] used Monte Carlo methods to simulate differences in the degradability and crystallinity of polymers in biodegradable microparticles for injectable levonorgestrel contraceptives. The contribution of drug diffusion and polymer degradation was evaluated, and the predictive power of the machine learning models was validated. Pishnamazi et al. [104] developed a hybrid ANN–Kriging method to simulate the release rate of aspirin from controlled tablets prepared using lignin and the dry granulation method. The Kriging interpolation method was used to increase the number of training and validation points for the ANN.

### 3.2. Estimation of Drug Dissolution

As discussed above, drug release and dissolution can be governed by a variety of mechanisms, depending on the formulation factors and drug properties, and this may markedly influence drug absorption. For this reason, the determination of drug dissolution (or drug release rate) has been a priority in different stages of formulation development. In terms of modeling drug dissolution, the Noyes–Whitney model, an extension of Fick’s first law of diffusion, is specifically designed to explain the dissolution process of solid drug particles in a solvent. It integrates the principles of diffusion characterized by Fick’s law to model the rate at which a drug dissolves, taking into account the diffusion coefficient, the concentration gradient, and the surface area of the dissolving solid.

When it comes to PBBM, the main interest lies in the estimation of drug dissolution in vivo. This process cannot be quantitatively described in vivo, so the options are to predict drug dissolution based on the properties of the drug and formulation or to measure it in vitro. In the latter case, the choice of in vitro experimental conditions is essential for the relevance of the dissolution test results. For example, if the drug is poorly soluble and ionizes in the physiological pH range, the physiological conditions will have a significant impact on the dissolution of the drug, which means that the in vitro setup should closely mimic the in vivo environment. In this context, one should consider two definitions from the regulatory guideline [105]. The first one, in vitro bio-predictive dissolution testing, refers to a set of conditions for which in vitro dissolution profiles are capable of predicting drug concentration profiles in plasma. These conditions are usually selected based on the results of the in vitro–in vivo correlation (IVIVC), which means that there is a correlation between the in vitro and in vivo dissolution profiles, but the conditions in vitro and in vivo do not necessarily have to be the same. Another assumption regarding bio-predictive dissolution testing is that similar dissolution profiles of drugs in vivo result in similar plasma levels, meaning that the in vitro test is able to reveal differences between the products that would be clinically relevant. The other term refers to a bio-relevant dissolution test, which represents a set of conditions designed to resemble a relevant physiological environment. The choice of bio-relevant experimental conditions depends on drug properties, dosage form characteristics, and physiological conditions at the site of drug release/dissolution. Bio-predictive and bio-relevant in vitro dissolution test conditions have been much discussed over the years, and further details can be found in the relevant literature [106,107,108,109,110,111,112,113,114,115]. It is also interesting to note that nowadays, several software packages are available to simulate the dissolution of drugs under different conditions, such as DDDPlus [18], DDSolver [116], or SIVA toolkit [117]. These software tools can greatly facilitate and accelerate the selection of the in vivo relevant conditions for in vitro testing, as demonstrated in several publications [118,119,120,121,122]. However, these tools are still being improved and their potential is not yet sufficiently exploited.

## 4. In Silico Modeling of Oral Drug Permeation and Absorption

There are two ways to look at the in vitro dissolution profiles in relation to PBBM. On the one hand, in vitro dissolution data can be used as inputs for the simulation (if obtained under bio-predictive or bio-relevant conditions) to estimate the resulting absorption and plasma concentration profiles. On the other hand, PBBM can be used to simulate drug dissolution and estimate the influence of drug-, formulation-, or physiology-related factors on this process to clinically relevant dissolution specifications. The link between bio-predictive or bio-relevant dissolution testing and PBBM and the utility of PBBM in terms of identifying in vivo relevant dissolution testing conditions or issues related to drug dissolution have been addressed in a number of publications [123,124,125,126,127,128,129,130,131,132].

The concomitant step that determines oral drug absorption after dissolution is permeation. For a drug to enter the bloodstream, it must cross at least one biological membrane. The epithelial cell layer in the intestine, the most important absorption site for orally administered drugs, has a specific structure that is described in simplified terms as a lipid bilayer with embedded proteins and is interrupted by tight junctions between the cells. Drug transport across the membrane is limited to several possibilities, depending on the properties of the drug, either through the lipid membrane (transcellular) or through the aqueous pores between the cells (paracellular). Each of these processes has specific features and can be described mathematically within a PBB model, as summarized in Table 5.

The total drug permeability reflects the overall drug transport via different mechanisms. This value can be determined experimentally using various methods (Table 6) or predicted using computer tools [133,134] and used as the input for PBBM. Further details on the available models for studying drug transport across the intestinal epithelium can be found in several comprehensive reviews [135,136,137,138,139,140].

The value corresponding to a human PBB model is the value determined in human subjects, but these data are rarely available as permeability testing in humans is not routinely performed. Alternatively, the values determined, e.g., in cell cultures or animal models, can be converted to human permeability based on a correlation between two datasets for a representative selection of reference compounds for which human permeability values are available [142,143]. In such cases, the selected reference compounds should have different permeabilities, from low to high, and the experimental data for the conversion must be obtained in the same laboratory and with the same method. Although the permeability conversion approach can be quite useful for PBBM, it should be used with caution as the values obtained in non-human models may not account for all permeation mechanisms involved in drug transport through the human intestine (e.g., PAMPA assays are only indicative of passive diffusion, expression of transporters in cell-based methods or animal models may not correspond to the expression in humans) [144,145,146,147]. In addition, drug permeability may depend on the region of the GI tract, and these variations can either be considered as region-dependent input values [148,149,150] or predicted based on drug permeability in a single region and parameters reflecting GI tract characteristics [5,151,152].

The estimated drug permeability data, together with the drug dissolution rate, are eventually used to assess the rate and extent of drug absorption. Absorption has been described by a variety of theories and equations over the years, as outlined in a number of reviews [16,153,154,155,156]. Most PBB models use compartmental absorption and transit (CAT) models, such as the advanced compartmental absorption and transit (ACAT) model within GastroPlus^TM^ software [151] or the advanced dissolution, absorption, and metabolism (ADAM) model within Simcyp™ PBPK Simulator [157]. These models interpret the gastrointestinal tract as a system of interconnected, sequential compartments described by a variety of physiological parameters that are mapped in dynamic equations to estimate drug transit, dissolution, and absorption. ACAT and ADAM models also take into account the possible metabolism of the drug in the gut wall, precipitation in the GI, and luminal degradation. In addition to CAT models, PK-Sim^®^ software is based on the “modified convection–dispersion model”, which treats the human intestine as a single tube, but with different properties in different regions and continuous intestinal transit function [158]. The comparative evaluation of this software has been addressed in several publications [5,159,160]. 

In general, all these models are able to predict drug bio-performance and mechanistically explain the processes that a drug and a dosage form undergo in the GI tract, but the criteria for estimating PBBM prediction accuracy are not clearly defined [161,162,163,164]. In addition, their predictive power depends on the complexity of in vivo processes and interactions between drug properties and physiology, as well as their interpretation in a PBB model. This means that any improvement in our knowledge of the in vivo processes governing drug absorption or mathematical equations that quantitatively describe these processes will inevitably improve the performance of PBB models. Moreover, the choice of input values markedly influences PBBM results, which means that increasing confidence in the input data would also lead to more reliable prediction results.

## 5. In Silico Modeling of Percutaneous Drug Permeation 

The skin is not only a unique physiological barrier but also an important route of drug administration to achieve a local or systemic therapeutic effect. Topical application of the drug ensures direct contact of the active pharmaceutical ingredients (APIs) with the diseased skin and minimizes the risk of side effects, while application of the drug to the intact skin for transdermal delivery is a simple and painless option that offers the possibility of prolonged drug release as well as reduced frequency of application and fewer side effects compared to peroral and parenteral routes. The main challenge in applying drugs to the skin is the permeation of the active ingredient to the site of action in the skin or bloodstream at the effective concentration. There are three generally recognized percutaneous permeation routes: (1) through the continuous stratum corneum, (2) through the hair follicles with the associated sebaceous glands, and (3) through the sweat ducts [165]. The percutaneous permeation processes are not yet fully understood, and it is usually assumed that the intact stratum corneum—the outermost layer of the skin epidermis consisting of dead, flattened, protein-rich cells (corneocytes) arranged like building blocks and connected by a complex lipid matrix between the corneocytes—is, paradoxically, the main route and rate-limiting step for the permeation of APIs. In addition, skin permeation depends on many factors, including the concentration and physico-chemical properties of the permeating drug and pharmaceutical excipients with permeation-promoting activity, skin characteristics (age, anatomical location, injury, disease), and environmental conditions (e.g., humidity, temperature) [166]. Therefore, the consideration of percutaneous permeation is one of the key aspects in the discovery and development of topical and transdermal drug delivery systems and, at the same time, an extremely challenging task. It can be studied experimentally using various in vitro methods (usually using the Franz diffusion cell with excised skin from humans, pigs, hairless rodents, guinea pigs, or artificial membranes) and in vivo (on various animal models). The experimental methods are well accepted, but there are ethical issues, inter- and intra-individual variation, interlaboratory differences with limited correlation, and the complexity of transferring data obtained on animal models to human skin [167,168,169]. Therefore, there is a growing need for validated and satisfactory alternatives to elucidate skin permeation in the early phase of drug development.

The field of computational (in silico) methods for the description and prediction of percutaneous permeability has been intensively developed for several decades. The in silico approach is promising for overcoming the limitations of in vitro, ex vivo, and in vivo permeability measurements in terms of ethical aspects that must be respected and insufficiently harmonized experimental protocols and conditions (i.e., intra- and inter-laboratory variability), which makes it difficult to evaluate the results of different studies. Numerous in silico methods (mathematical models and computer simulations) have been proposed by various scientists and research groups as a cost-effective and less time-consuming complement or alternative to experimental permeation assessment, as well as to predict the permeation of known substances, newly synthesized molecules, or not yet synthesized molecules [170,171,172]. 

**Figure 4:**
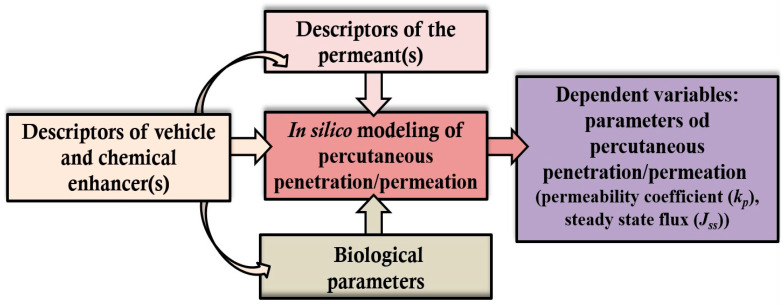
Schematic representation of the interplay of descriptors (independent variables) for in silico modeling of percutaneous permeability.

The comprehensive reviews of in silico modeling of percutaneous penetration, permeation, and absorption of the entire spectrum of chemical substances have been extensively considered [173,174,175,176,177,178]. The models used are empirical (based on experimental data) or mechanistic (based on physical and biochemical principles). Mechanistic models are particularly useful in analyzing the mechanism of the permeation process of actives relevant to human skin, as well as in studying the permeation of actives in the presence of permeation enhancers, which facilitates the design of topical/transdermal formulations. The most commonly used models to date are quantitative structure–permeation relationship (QSPR) (also quantitative structure–activity relationship (QSAR)) and molecular dynamics (MD) simulations [179,180]. The interplay of descriptors for in silico modeling of percutaneous permeability is represented in Figure 4.

### 5.1. QSPR/QSAR Models

QSPR/QSAR models describe a linear or non-linear mathematical relationship of percutaneous permeation based on experimental data (empirical QSPR models) or diffusion of the permeate at steady state (mechanistic QSPR models) with its molecular structural properties and physico-chemical characteristics (descriptors) [179,181,182,183]. Linear QSPR/QSAR models are usually based on multiple linear regression (MLR), partial least squares (PLS), linear free-energy regression (LFER), and principal component regression (PCR) [180]. Linear QSPR/QSAR models can provide reliable insight into the relationship between percutaneous permeation and permeant descriptors such as lipophilicity and molecule size. However, when the relationships between skin permeability and a variety of descriptors are more complicated, nonlinear models based on machine learning perform better. The choice of a QSPR/QSAR model depends on the expected responses, and the predictions should be reliable [179]. QSPR/QSAR modeling enables the elucidation of the influence of physico-chemical properties of permeants on permeation at the molecular level, as well as the identification of permeants with challenging physicochemical properties or the prediction of target properties of permeants that could be promising candidates for experimental evaluation, thus facilitating the screening of chemical databases of newly synthesized compounds or virtual libraries (prior to their synthesis). Many QSPR/QSAR models are available in various computer software packages. Data obtained in mixed animal and human experiments, different vehicles, on various skin sample regions, and under different measurement conditions can be used to develop software [180]. Although the QSPR/QSAR approach allows a relatively fast evaluation of large datasets, the in silico predicted skin permeability depends more or less on the quality of a software database and may vary between different models.

For the steady-state permeation of permeable molecules through the lipid matrix of the stratum corneum, based on the passive diffusion process from a skin region of high concentration to low concentration, the QSPR approach usually predicts two parameters—the steady-state flux (*J_ss_*) and the permeability coefficient (*k_p_*). According to Fick’s first law of diffusion at steady state [184], when the permeant concentration is kept at a constant value and the maximum solubility of the solute in the stratum corneum is reached, and assuming that the stratum corneum is a pseudo-homogeneous membrane whose barrier properties do not change with time, *J_ss_* can be defined as the permeation rate (the amount of permeant per unit surface area of exposed skin and time) (dimensions of μg/cm^2^/h). Here, *k_p_* is *J_ss_* normalized by the concentration gradient (i.e., *J_ss_* divided by the concentration of the applied penetrant (typically in a saturated aqueous solution)) (dimensions in cm/h or cm/s). Therefore, *J_ss_* is suitable for comparing a wide range of permeants from saturated solutions, whereas *k_p_* depends on the diffusion coefficient, the skin-to-vehicle partition coefficient of the permeant, and the path length [170,179].

In the early 1990s, Potts and Guy developed the first QSPR model [185] based on human skin permeability coefficients for 97 drug substances from aqueous solutions compiled from 15 different literature sources and published by Flynn [186]. The Potts and Guy model (PGM) is the most frequently cited and applied empirical linear QSPR model for predicting skin permeability (*logk_p_*) based on known physico-chemical properties of a permeant–water–octanol partition coefficient (*logP*) and molecular size (in terms of molecular volume or molecular weight (*MW*)):

(18) $$logk_{p}=-6.3+0.71logP-0.0061MW$$

where *k_p_* is in cm/s, *MW* ranges from 18 to 750, and *logP* ranges from −3 to +6.

The PGM is used by many regulatory agencies and the U.S. Environmental Protection Agency (EPA) as a valuable tool for assessing potential systemic absorption from skin exposure [187]. The PGM assumes that the lipophilic stratum corneum is the rate-limiting factor for skin permeation, meaning that increased lipophilicity (lower water solubility) and smaller molecule size increase skin penetration. So far, *logP* and *MW* are the two most commonly used descriptors correlated with skin permeability. However, it follows that this model cannot be used when formulation components modulate the barrier properties of the skin. Furthermore, the PGM assumes simple aqueous solutions of permeants, so in some cases, the extrapolation of predictions to complex multicomponent and/or multiphase formulations is unclear. Although the PGM has often been considered more accurate compared to some newer and more complex mathematical models, in some cases, it over- or under-estimated transdermal flux [188]. The inclusion of molecular volume, polarizability, hydrogen bond donor and acceptor activities, and molar refractive index improved the fit of the PGM to more limited datasets [189,190].

Several research groups have developed related QSPR models and a large number of skin permeability coefficients for a range of permeants based on extensive databases [190,191,192,193,194,195], indicating the importance of selecting the molecular structure (rather than lipophilicity) of the permeant, physico-chemical properties of the vehicle and the biological system/membrane used. Table 7 represents an overview of in silico modeling for the prediction of percutaneous permeability from different permeant descriptors.

Cronin et al. [190] generated the QSPRs based on the literature data using least-squares regression analysis. The QSPRs were used to calculate 47 descriptors from the relevant physico-chemical parameters of 114 drugs and to predict and compare their permeability coefficients through excised human skin in vitro and through a synthetic polydimethylsiloxane membrane. The hydrophobicity and molecular size of the penetrant influenced the percutaneous absorption. The mechanisms of drug permeation through the studied membranes differed significantly. Chang et al. [191] created the QSAR model for 158 chemical substances with known permeability coefficients using 4 molecular descriptors (the electrostatic interactions between the electric quadrupoles of van der Waals forces, the octanol–water partition of the solute, the similarity of antineoplastic property, and the abundance of carbon–nitrogen bonds at a constant topological distance) to relate the physico-chemical properties and transdermal transport of a permeant. Patel et al. [195] developed QSARs using hydrophobicity, molecular size, and hydrogen bonding as descriptors to determine the skin permeability coefficients of 158 compounds through excised human skin in vitro. Although the descriptors provided an excellent fit to the data (r^2^ = 0.90), the permeability data for many compounds (e.g., hydrocortisone derivatives) were erratic and were therefore removed from the dataset. Magnusson et al. [196] suggested that for data analysis of permeants from water vehicles using the QSPR method, *J_ss_* is, practically, a more relevant parameter than *k_p_*. Also, *logP* appeared to be a less significant parameter to improve predictions, and *MW* alone was sufficient to describe most of the chemistry-specific variations in the data, according to the rule that a *MW* below 500 Daltons is necessary for permeate permeation through the skin.

The distribution of permeant between water and the stratum corneum is expected to have a correlation with its lipophilicity, but this may not be trustworthy in some cases. An empirical QSPR model was constructed by Liou et al. [197] to predict the permeability coefficients of 13 non-steroidal anti-inflammatory drugs (NSAIDs) by considering the solubility parameter (*δ*) of those model drugs, assuming the penetration of drugs with *logP* > 2 and <2 via hydrophobic (nonpolar) and hydrophilic (polar) pathways, each of which would encounter different properties determined by the biological parameters (transepidermal water loss (TEWL), hydration content (HD), lipid content (SB), resonance running time (RVM), and elasticity (EL)) of the individual skin samples. The drug characteristics (*MW*, polarity factor (*logP*), and *δ*) and the measured biological parameters were the independent variables for the construction of the QSPR model. The regression procedure (stepwise function) in SAS 8.0 statistical software was used to obtain the relationship between the independent variables and the dependent variable (*k_p_*). In vitro permeation of NSAIDs through the full-thickness skin of nude mice was investigated. The predictive ability of the developed QSPR model was demonstrated by validating a plot of the observed *k_p_* values against the predicted *k_p_* values. The study showed that the QSPR model could be statistically improved by incorporating *δ* and biological parameters and that *δ* could be a more appropriate drug descriptor for predicting *k_p_* of those NSAIDs with *logP* < 2 with an adjusted R > 0.90, compared to the PGM. The solubility parameter (*δ*) is proposed as a possible replacement for the partition coefficient in the evaluation of skin permeability when a solute is distributed between the stratum corneum and the water phase.

Several studies have compared the predictive ability and accuracy of the PGM with other QSPR models using different datasets. Lian et al. [198] compared 7 models using skin permeability data of 124 chemical compounds from different sources and concluded that the PGM provided the second best predictions, while the best predictions were offered by the mechanistic model of Mitragotri [199]:

(19) $$logk_{p}=0.7logP-0.0722MW^{2/3}-5.2518$$

Both the PMG and Mitragotri models assume diffusion of the permeant through the lipid matrix and predict skin permeability based on the two physicochemical properties (*logP* and *MW*). In addition, the Mitragotri approach was derived to predict *k_p_* by using a scaled particle theory for solute partitioning and considering diffusion through a lipid bilayer as an isotropic phase with a tortuosity of *τ*:

(20) $$k_{p}=5.6\times 10^{-6}P^{0.7}\exp\left(-0.1662MW^{2/3}\right)$$

**Table 7:** The representative references demonstrating the applicability of in silico modeling for the prediction of percutaneous permeability (logkp or flux) from different permeant descriptors.

In Silico Model	Considered Descriptors	In Silico Evaluated Percutaneous Permeability	References
QSPR	*logP* and molecular size (*MV* or *MW*)	*logP* and *MW* of a single permeant from aqueous solution correlated well with *logk_p_*	[185,199]
QSPR	47 descriptors from relevant physico-chemical parameters of 114 drugs	Hydrophobicity and the molecular size of the penetrant affected *logk_p_*	[200]
QSPR	Lipid solubility of 13 corticosteroids and sex steroids	The predicted flux of steroids was not accurate	[201]
QSPR	*MV*, *logP*, and *MP*	The accuracy in the predicted permeability *logk_p_* was demonstrated with 60 molecules, including small molecules and steroids	[181]
QSAR	Hydrophobicity, molecular size, and hydrogen bonding of 158 compounds	The descriptors provided an excellent fit to the permeability data for most compounds except hydrocortisone derivatives	[195]
QSPR	*logP*, *MW*, *MV*, and the melting point of betamethasone dipropionate, clobetasol propionate, fluorouracil, flurandrenolide, ketoconazole, lidocaine, metronidazole, tacrolimus monohydrate and tazarotene (formulated in propylene glycol and commercial formulations)	The QSPR models were useful for skin permeability assessment, although discrepancies were observed for tazarotene, tacrolimus, ketoconazole, and metronidazole	[200]
QSPR	*MW*, *logP*, and *δ* (assuming the penetration of drugs with *logP* > 2 and <2 via nonpolar and polar pathways, respectively) of 13 non-steroidal anti-inflammatory drugs (NSAIDs), and biological parameters (*TEWL*, *HD*, *SB*, *RVM*, and *EL*) of individual skin samples	Inclusion of *δ* and biological parameters improved statistically the QSPR model for predicting *logk_p_* of NSAIDs with *logP* < 2	[197]
QSPR/QSAR integrated with molecular docking	*MW*, *MV*, predicted *logP*, total polarity surface, and hydrogen bond of the phytosterols (campesterol, β-sitosterol, and stigmasterol)	The predicted *logk_p_* was the greatest for β-sitosterol, followed by campesterol and stigmasterol. The in vivo study (mice) confirms the capacity of topically applied β-sitosterol as an antipsoriatic agent	[182]
QSPR/QSAR integrated with molecular docking	Molecular size (the number of resveratrol subunits) and physico-chemical properties (MV, *logP*, hydrogen bond (H-bond) number, and total surface polarity) of resveratrol and its oligomers	Oligomers with higher numbers of subunits have higher docking scores and are predicted to bind stratum corneum lipids more strongly; ε-viniferin was identified as a promising antipsoriatic agent that accumulated at higher levels in psoriasis-like mouse skin	[183]
QSAR	*logP* of the drug (haloperidol) and descriptors of 49 terpenes (*MW*, *logP*, boiling point, melting point, the terpene type, and the functional group of each enhancer)	The ideal terpene enhancer for haloperidol has at least one or combinations of the following properties: larger *logP*, liquid state at room temperature, with an ester or aldehyde (but not acid) functional group, and is neither a triterpene nor tetraterpene	[193]
Membrane-interaction QSAR (MI-QSAR)	MI-QSAR descriptor (the difference in the integrated cylindrical distribution functions over the phospholipid monolayer model, in and out of the presence of the skin penetration enhancer (ΔΣh(r)) developed for two datasets of 61 and 42 penetration enhancers	Explained 70–80% of the variance in skin penetration enhancement across each of the two training sets to predict skin permeability enhancement for hydrocortisone and hydrocortisone acetate	[202]
QSPR	Donor/acceptor interactions, van der Waals forces, HBD–π interactions, and hydrogen bonding in complexes of four APIs (5-fluorouracil, hydrocortisone, estradiol, and diclofenac sodium) and 34 terpenes	The satisfactory correlation between the predicted molecular properties of modeled complexes or examined terpenes and the permeation enhancement effects	[203]
ANN-based QSAR	*logP*, *MW*, steric energy, van der Waals area, van der Waals volume, dipole moment, highest occupied molecular orbital, and lowest unoccupied molecular orbital of 35 newly synthesized O-ethylmenthol derivatives	*logP*, steric energy, and the lowest unoccupied molecular orbital significantly affected the prediction of ketoprofen enhancement factor (penetration rate with enhancer:penetration rate without enhancer) (Ef) and total irritation score (TIS)	[204]
ANN and RSM	Vehicle composition (water (W), ethanol (E), propylene glycol (P), their binary and ternary mixtures)	RSM and ANN coincided very well in the prediction of the most suitable mixtures (W:E:P (20:60:20), W:E (40:60), and W:P (50:50)) to increase flux and reduce lag time of percutaneously applied melatonin	[205]
ANN and differential evolution (DE))	Statistically significant descriptors of potential permeability enhancers of insulin included: average 1-electron reactivity index for a carbon atom, minimum 1-electron reactivity index for an oxygen atom, Kier and Hall index (order 1), RNCS relative negative charged SA (SAMNEG*RNCG) [Zefirov’s PC], and total dipole of the molecule.	The compounds with greater hydrophobicity and reactivity, as well as low dipole moments and capacity to form intermolecular bonds with stratum corneum lipids, could be promising insulin-specific permeability enhancers	[206]

*logP*—partition coefficient between water and octanol; *MV*—molecular volume; *MW*—molecular weight; *δ*—solubility parameter; *TEWL*—transepidermal water loss; *HD*—hydration content; *SB*—lipid content; *RVM*—resonance running time; *EL*—elasticity; *MP*—the solubility of the permeant in both polar and non-polar solvent.

Alonso et al. [200] evaluated the skin permeability of 20 marketed topical drugs using the PGM and Barrat model (BM) [181] and an in vitro assay with an artificial membrane (Skin-PAMPA). The BM uses a linear PCR approach and considers the molecular volume (*MV*) instead of the *MW*, as well as the solubility of the permeating agent in polar and non-polar solvents (*MP*) as additional parameters for the calculation of the skin permeability coefficient (*logk_p_*):

(21) $$logk_{p}=0.82logP-0.0093MW-0.039MP-2.36$$

The accuracy of the predicted permeability of the BM was demonstrated with 60 molecules, including small molecules and steroids [181]. In addition, in the study by Alonso et al. [200], 9 APIs formulated in propylene glycol (PG) and commercial formulations (betamethasone dipropionate 0.5 mg/g cream (Diproderm), clobetasol propionate 0.5 mg/g cream (Clovate), fluorouracil 50 mg/g cream (Efudix), flurandrenolide 0.5 mg/g cream (Cordran), ketoconazole 20 mg/g cream (Fungarest), lidocaine 20 mg/g cream (Dermovagisil), metronidazole 7.5 mg/g gel (Rozex), tacrolimus monohydrate 1 mg/g ointment (Protopic), and tazarotene 1 mg/g gel (Zorac)), were tested in Franz cells using human or porcine whole skin samples. The APIs were classified according to their determined/predicted skin permeability. The aim of the study was to translate different physico-chemical properties of the APIs into a wide range of skin permeabilities to enable the prediction of skin permeability using the in silico and in vitro models. The physico-chemical properties (*logP*, *MW*, and *MV*) were calculated for all APIs, and the melting point was measured. The mathematical models studied were comparable and useful for the evaluation of skin permeability, although discrepancies were observed for some drugs (tazarotene, tacrolimus, ketoconazole, and metronidazole), which can be explained by the differences in the methods and the variety of physico-chemical properties of the compounds.

Burli et al. [188] investigated the ability of the PGM and Cleek and Bunge´s model (CBM) [201] to predict the transdermal flux of 13 corticosteroids and sex steroids (estradiol, progesterone, fluocinolone acetonide, dexamethasone, cortisone, corticosterone, desoxycortone, dehydroepiandrosterone, androstenedione, testosterone, hydrocortisone, 17-OH progesterone, and testosterone acetate) and their accuracy is compared with previously published in vivo data on percutaneous absorption following topical administration of the steroids dissolved in acetone in Caucasian individuals (ages 21–50) [207]. The PGM was used to predict the permeability coefficient (*k_p_*). In vivo flux (*J_ivv_*) was calculated from previous experimental in vivo data as follows:

(22) $$J_{ivv}=maximal\;absorption\;rate\times applied\;dose$$

the maximal absorption rate was in %/hour, whereas the applied dose was 4 μg/cm^2^ (4000 ng/cm^2^).

The CBM takes lipid solubility into account and has been shown to be more suitable for lipophilic substances than the PGM. The PGM correlated significantly with the in vivo data, but the flux of most steroids studied was overestimated (by a factor of 2.5 up to a factor of 60) and, in one case (testosterone acetate), underestimated (by a factor of 5). The observed statistical differences between the predicted and in vivo derived results could be related to the following factors: anatomical variations (variations in the amount of stratum corneum and skin thickness), rubbing and washing of the skin (washing-in and washing out), shunt diffusion (diffusion involving hair follicles, sebaceous and sweat glands), age differences, variability of PGM parameters between laboratories, variability of in vivo data, sample size (only 13 steroids). In addition, the PGM was developed for the evaluation of percutaneous penetration of chemical substances in water-based vehicles and under steady-state conditions, while the in vivo study used steroids in acetone and finite single-dose kinetics. CBM fitting for *k_p_* may allow better predictability than PGM due to the low water solubility of steroids. The general observation of the study was that the flux of steroids predicted by the mathematical models considered was not accurate, probably because they do not take into account volatility, lipid solubility, hydrogen bonding effects, drug metabolism, and protein binding aspects.

The need to correlate skin permeability with a variety of permeation descriptors and parameters that account for the effects of different solvents/delivery vehicles on the permeation process has encouraged the development of non-linear SPQR models, using machine learning algorithms such as artificial neural networks (ANN), neural fuzzy algorithms, decision trees, decision forests, random forests, support vector machines, Gaussian regressions, k-nearest-neighbor regression, ridge regression, and conformal prediction [179,180]. In addition to the descriptors from the PGM, some of the descriptors considered in nonlinear QSPR analysis are permeant solubility, dipole moment, polarizability, solvation free energy, number of hydrogen acceptor and donor bonds, the sum of the charges of the nitrogen and oxygen atoms and the sum of the charges of the hydrogen atoms bound to nitrogen or oxygen atoms, as well as the octanol–water partition coefficient measured at a certain pH value. Numerous studies have been published over the last three decades, often demonstrating the superiority of non-linear models over linear models. Nevertheless, a number of challenges have been recognized that currently limit the applicability of non-linear models, including the tendency to overfit and model the error inherent in the data, as well as limited mechanistic insight. The need to develop and apply specialized expertise and/or programs for data processing and correct interpretation is often criticized [170]. In a very recent study, Vidović et al. [208] applied various QSPR statistical models available online to predict percutaneous permeability in a group of 24 newly synthesized succinimide derivatives (1-aryl-3-methylsuccinimides (Series C) and 1-aryl-3-ethyl-3-methylsuccinimides (Series D)) with antibacterial and antifungal activity. Skin-PAMPA was also performed using an isopropyl myristate/silicone oil mixture (3:7) membrane and PBS buffer solutions with a pH of 7.4 in the donor and acceptor sections of the MultiScreen Trans-port Receiver Plate (Millipore, Burlington, MA, USA) to determine the apparent permeability coefficient (Papp-skin). The determined Papp-skin values were compared with the in silico predicted skin permeability and lipophilicity using three different prediction models: the free online tool SwissADME [209] (to calculate logarithm of the partition coefficient (iLogP, XLOGP3, WLOGP, MLOGP, SILICOS-IT logP, and Consensus LogP) and *logk_p_* (cm/s)) and the program pkCSM [210] and the PreADMET online server [211] (to calculate *logk_p_* (cm/h)). Data classification analysis was performed with the statistical techniques of principal component analysis (PCA) and hierarchical cluster analysis (HCA) using the program Statistica v.12 (StatSoft Inc., Tulsa, OK, USA, 2012). The sum of ranking differences (SRD), a non-parametric method introduced by Héberger and Kollár–Hunek, was used to rank the analyzed compounds in terms of their in silico lipophilicity and percutaneous permeability as well as in vitro estimated percutaneous permeability. All compounds are considered to have relatively good skin permeability at a pH of 6, while 2 compounds containing carboxyl groups attached to the main core were found to ionize and have limited permeation at a pH of 7.4. A statistically significant correlation was found between the in silico predicted *logk_p_* values and the Papp-skin values, as well as between Papp-skin and the calculated *logP* data. PCA, HCA, and SRD data analysis of the in silico *logk_p_* and calculated *logP* data revealed that lipophilicity is an important (but not the only) physico-chemical characteristic for passive percutaneous permeation of the succinimide derivatives studied. Although the combination of mathematical predictions and experimental assessment of skin permeability provides a clearer insight into the probability of percutaneous permeation of a molecule and thus offers a screening of potential bioactive compounds for cutaneous application, it does not allow insight into the permeation mechanism.

With the aim of overcoming the limitations of single linear and non-linear approaches, the recent study by Wu et al. [212] predicted the skin permeability coefficient and investigated the intrinsic permeation mechanism using a novel two-QSAR approach [213] comprising machine learning-based hierarchical support vector regression (HSVR) [214] and linear PLS. A compilation of published data on the permeability of 96 compounds from human skin sampled ex vivo was generated. There was an apparent bi-linear relationship between *logk_p_* and *logP* (i.e., *logk_p_* initially increased with *logP* and then decreased at *logP* ≥ 4), which cannot be properly captured by linear models alone, so the non-linear machine learning approach was included. The mock test was performed to calibrate the derived HSVR and PLS models to the compounds studied by Soriano–Meseguer et al. [215], of which 23 were included. The HSVR model obtained a better association between the measured *logP* and the predicted *logk_p_* than PLS, while the PLS model showed relevance for the interpretation of skin permeation mechanisms. Furthermore, PLS and HSVR were compared with the skin permeation calculator (SPC) [216] developed by the U.S. Center for Disease Control and Prevention (CDC). SPC was applied to the 96 compounds studied, and skin permeation could be predicted for 80% of the compounds, showing limited applicability compared to PLS and HSVR. The study recommends the synergistic use of predictive HSVR and interpretable PLS models to facilitate drug discovery and development by predicting skin permeability.

Recent studies show the integration of molecular docking with the QSPR method. Chang et al. [182] investigated QSPR/QSAR for the phytosterols (campesterol, β-sitosterol, and stigmasterol) to evaluate how their physico-chemical properties affect their skin transport and ability to alleviate psoriasiform inflammation. In silico molecular modeling of the physico-chemical properties and molecular docking of phytosterols to stratum corneum lipids (ceramides, palmitic acid, cholesteryl sulfate, and cholesterol) was performed. The physico-chemical properties of the phytosterols (*MW*, *MV*), predicted *logP* value, total polarity surface, and hydrogen bonding (H-bonding)) were estimated from the molecular structures outlined using Discovery Studio 2021 (Dassault Systems, Paris, France). Docking simulation was applied to measure the negative CDOCKER energy between the phytosterols and the lipids of the stratum corneum, reflecting the bond strength. The in silico predicted skin permeability (*logk_p_*) of three phytosterols was calculated using an online server [217]. The in vitro permeation test on excised porcine skin was performed according to OECD 428 guidelines [218]. The tested phytosterols possessed an H-bond acceptor and an H-bond donor and showed high lipophilicity. The values of the oil/water partition coefficient calculated using molecular modeling and the predicted partition coefficient were highest for β-sitosterol. The highest skin permeability was predicted for β-sitosterol, followed by campesterol and stigmasterol. The anti-inflammatory activity of the phytosterols in the activated keratinocytes was comparable, but a phytosterol structure without a double bond at C22 and with methyl moiety on C24 (campesterol) was more effective in the macrophage-based study. The order of absorption by porcine skin was β-sitosterol (0.33 nmol/mg), campesterol (0.21 nmol/mg), and stigmasterol (0.16 nmol/mg). The therapeutic index was determined by multiplying the percentage of cytokine/chemokine suppression by skin absorption, and the highest value was recorded for β-sitosterol. The in vivo animal study in mice confirms the beneficial effect of topically applied β-sitosterol as a potentially potent antipsoriatic agent. Cheng et al. [181] demonstrated the usefulness of in silico molecular modeling and docking (Discovery Studio 4.1, Accelrys, San Diego, CA, USA) to investigate the influence of molecular size and physico-chemical properties of resveratrol glycoside (polydatin) and resveratrol oligomers (ε-viniferin (dimer), ampelopsin C (trimer), and vitisin A (tetramer)) on cutaneous absorption. In addition, in vitro tests (using the Franz cell and the porcine skin membrane) and in vivo tests (using imiquimod-treated mice) were performed to identify the promising antipsoriatic drug candidate for topical therapy. The effect of the number of resveratrol subunits on skin absorption was investigated to determine the QSPR. The physico-chemical properties of resveratrol and its oligomers (molecular volume, estimated lipophilicity (*logP*), number of hydrogen bonds (H-bonds), and overall surface polarity) were predicted using Discovery Studio 4.1. The compounds were superimposed on the stratum corneum lipids to estimate the ligand-binding interactions by calculated molecular docking and to identify possible interactions between the permeants and stratum corneum lipids. Negative CDOCKER values, indicating a stronger affinity between the permeant and lipids, were calculated by docking simulation. In silico modeling revealed that oligomers with a higher number of subunits had higher docking values and were more likely to bind to lipids due to the higher *MW* and H-bond numbers. Therefore, the monomers (resveratrol and polydatin) exhibited a higher flux through the lipids of the stratum corneum to viable skin than the larger oligomers. In contrast, the in vivo absorption of the oligomers, especially the tetramer (but not the monomers), was significantly increased in barrier-damaged skin. The study identified ε-viniferin as a promising antipsoriatic agent that accumulated at higher concentrations in psoriasis-like mouse skin, alleviated psoriasiform symptoms, and reduced hyperplasia and inflammation more effectively than resveratrol.

### 5.2. MD Simulations

MD simulation is a computer approach for simulating, recording, calculating, and analyzing the physical movements of interacting atoms and molecules. It is suitable for analyzing the dynamic interactions between different molecules and the evolution of complex systems. MD simulations, as an experimentally independent approach for the prediction of percutaneous permeability, offer the possibility to recreate a skin barrier model and tend to increase the number of variables considered [180]. Although MD simulations trained without experimental data are used to predict permeability coefficients, a correlation between calculated and experimental results is required to verify the usefulness of the MD model used. Furthermore, the MD simulations can assist in the interpretation of in vitro skin permeability data. The MD simulations of the stratum corneum lipids are based on the well-characterized forces between atoms and molecules, which allow a simulation of their collective behavior.

The MD simulations of the structure and dynamics of the skin lipid barrier, permeation of different molecules across simple model membranes, and penetration enhancement mechanisms of chemical permeation enhancers (e.g., DMSO, ethanol, and oleic acid) are reviewed in detail elsewhere [180,219]. Several research groups have shown that MD simulations have the potential to provide insights into the process of interaction between different permeants and the simulated lipid membranes and a better understanding of their permeability through the biological membrane at the molecular level [220,221,222]. The reliability of MD simulations to predict the permeability of active compounds based on the knowledge of the molecular structure of the permeant is based on the understanding of the molecular organization of the skin barrier. The greatest attention is paid to the composition and architecture of the human stratum corneum [223].

The current application of MD methodology is limited by the heterogeneity of the composition of the intercellular lipids of the stratum corneum and by insufficiently understood aspects of the physical state of lipids within the barrier. As the current MD simulation approach is mainly focused on the stratum corneum and not on the whole skin, the applicability of this in silico strategy to elucidate the permeation process through the viable epidermis and dermis has not been sufficiently explored [224].

An important aspect of percutaneous permeation that can be tackled using MD simulations concerns the identification of the particular area or pathway of the skin barrier that represents the limiting step for the permeation process of a given API. Machado et al. [225] performed the MD simulation with a Martini force field to simulate the percutaneous transport of ascorbyl tetraisopalmitate, a hydrophobic and non-oxidizable vitamin C derivative, through the infundibulum and stratum corneum of the human hair follicle. MD simulation involves fine-grained (FG) simulation (i.e., simulation at the atomic scale where each atom is considered in the interaction process) and coarse-grained (CG) (simulation by grouping atoms) and considering four main types of the interaction sites: polar (P), nonpolar (N), apolar (C), and charged (Q). Further transformations were performed using the g_fg2cg algorithm implemented in the GROMACS 3.3.1 package in conjunction with the Gromos53a6 force field. The models of the infundibulum and the stratum corneum, including the realistic concentrations of the typical lipid components, were built with the program CELLmicrocosmos 2.2. For comparison, the two membrane models were compared, and the permeating molecules were placed between the two membranes in the same system. The models adopted took into account the increased permeation of hydrophobic substances within the infundibulum membrane, while the stratum corneum delays this permeation. This comparative study revealed that the ascorbyl tetraisopalmitate molecule has a higher affinity to the stratum corneum and, therefore, preferentially permeates this membrane, while in the infundibulum, it has a lower affinity and higher mobility, suggesting that permeation to deeper skin layers is more likely in the infundibulum than in the stratum corneum. It was indicated that the penetration of a single molecule required more time and was accelerated by increasing the permeation concentration. Moreover, this is the MD simulation where the specific thermodynamic conditions, such as pressure and skin temperature, were introduced, leading to a more accurate representation of the biological membrane under consideration.

MD simulations can provide insight into the mechanisms underlying the influence of chemical permeation enhancers, including the partition of the permeation enhancer in the barrier [226]. For this purpose, MD simulations are a good complement to other in silico and experimental methods.

### 5.3. In Silico Modeling of Skin Permeation in the Presence of Permeation Enhancers

Most in silico models are created to predict the passive skin permeation of a single permeant, often from an aqueous solution, and thus deviate from the real situation of in vivo administration of the dosage form (usually a mixture of one or more APIs and more than one pharmaceutical excipient) on the skin surface. For successful drug development, it is necessary to evaluate the permeability of APIs in relation to the composition of the vehicle, including the potential permeation-enhancing effect of the vehicle ingredients [200]. Li et al. [227] applied in silico molecular modeling and molecular docking methods to study the molecular interactions and explore the molecular mechanism of percutaneous absorption enhancement of sorbitan monooleate in vitro and in vivo for the optimized olanzapine transdermal patch. The geometry optimization of sorbitan monooleate, olanzapine, and the pressure-sensitive adhesive was performed by condensed-phase optimized molecular potentials for atomistic simulation studies (COMPASSII) force field, and the potential sites of hydrogen bonding interactions between the optimized structures were observed. The molecular modeling experiment showed the competition between the drug and the penetration enhancer in forming hydrogen bonds with the polymer. Sorbitan monooleate weakened the hydrogen bonding between olanzapine and the pressure-sensitive adhesive, thereby reducing the cohesive interaction between the polymer chains and promoting the release of olanzapine. Furthermore, molecular docking showed that sorbitan monooleate can interact with the polar head groups of the skin lipids (ceramides), increasing their fluidity and the percutaneous absorption of olanzapine.

The selection of already known and the design of new chemical permeation enhancers to facilitate percutaneous permeation of APIs is of great interest to the pharmaceutical industry [202]. The ongoing challenge is to clarify the influence of permeability enhancers due to the complex and probably multiple mechanisms that influence the permeation process. The results of experimental studies suggest at least three mechanisms: (1) enhancement of the API partitioning in the stratum corneum; (2) interactions of the enhancer with stratum corneum lipids leading to a disruption of their highly ordered structure and improving paracellular permeation; (3) interactions of the enhancer with intracellular proteins of corneocytes enhancing transcellular transport. In silico approaches expand the knowledge about the effect of chemical penetration enhancers on percutaneous permeation at the molecular level and provide evidence for the rational selection of such excipients. There have been several attempts to analyze the permeation processes in the presence of terpenes [228,229,230,231,232].

Kang et al. [193] used an MLR-based QSAR model to predict the activity of 49 terpenes and terpenoids, including monoterpenes, sesquiterpenes, diterpenes, triterpenes, and tetraterpenes with various functional groups such as hydrocarbons, alcohols, aldehydes, esters, ketones, and oxides, respectively, in enhancement of the in vitro permeability coefficients of haloperidol from a 5% solution in propylene glycol through excised human stratum corneum. The solubility of haloperidol in propylene glycol and *k_p_* were determined experimentally. The in vitro permeability test was performed using standardized experimental protocols in the same set of automated flow-through diffusion cells. In addition, *k_p_* was calculated using a nonlinear regression model. Haloperidol is a small hydrophobic molecule. Apart from the drug lipophilicity (*logP*), all the other predictors were qualitative variables. A first-order MLR model was used to construct a QSAR model linking *k_p_* to the *logP* and the descriptors of the terpenes (*MW*, *logP*, boiling point, melting point, the terpene type, and the functional group of each enhancer). The best regression model based on stepwise selection was:

(23) $$\log k_{p}=-9.13+0.344\log P+0.616Liquid-4.84Tri-7.37Tetra+2.03Aldehyde+1.49Ester-5.36Acid$$

where “Liquid”, “Tri” and “Tetra”, “Aldehyde”, “Ester”, and “Acid” are indicator variables standing for liquid terpene, triterpene, tetraterpene, aldehyde, ester, or acid functional groups, respectively.

Equation (23) indicates that terpenes with higher *logP* values are more effective enhancers than those with lower *logP* values, probably because they mix more easily with the stratum corneum lipids, and their extraction or transition reduces the barrier strength. At the same time, compounds with large *logP* permeate faster than those with small *logP* values. In addition, the ideal terpene enhancer should possess at least one or a combination of the following properties: hydrophobicity, liquid state at room temperature, with an ester or aldehyde functional group (but not acid), and is neither a triterpene nor a tetraterpene. Interestingly, the inclusion of *MW* and hydrogen bonding as common descriptors affecting the k_p_ of permeants did not improve the developed regression model. The authors proposed the developed QSAR model for the prediction of the skin permeation enhancement of APIs with similar physico-chemical properties as haloperidol by other terpene enhancers. Drakulić et al. [203] used a computational study of interactions of four APIs (5-fluorouracil, hydrocortisone, estradiol, and diclofenac sodium) and 34 terpenes to investigate their permeation enhancer influence. Molecular modeling indicated complexation via donor/acceptor interactions, van der Waals forces, HBD–π interactions, and hydrogen bonding between the APIs and the hydrocarbon and oxygen-containing terpenes, respectively, altering the physico-chemical properties of the APIs, as a possible mechanism to enhance penetration. The QSPR based on simple MLR achieved a satisfactory correlation between the predicted molecular properties of the modeled complexes or investigated terpenes and the permeation enhancement effects.

Several studies have addressed the application of ANN in predicting the permeability of APIs in the presence of permeation enhancers [179]. ANN is a machine-learning approach inspired by the structure of a neural network of the human brain, which consists of interconnected units (neurons). The mutual influence of the neurons is influenced by the values of the connections (weights). The architecture of ANN models varies from one- or two-layer unidirectional to complex multidirectional feedback layers. Although the ANN strategy is used to model processes that cannot be successfully considered with conventional modeling approaches, ANN models also have certain limitations. The experimental design and data collection are time-consuming, and ANN models are not suitable for elucidating the mechanistic aspect of the observed correlation. No special computers are required to obtain a reliable ANN model, but the user must have the necessary expertise and sufficient experience in the field of ANN modeling [233,234]. Nevertheless, there are examples in the literature of empirical ANN models that have shown useful and accurate prediction of skin permeability based on physico-chemical properties (*MW*, *logP*, Abraham descriptors) of numerous compounds, including APIs [235,236,237]. For example, Degim et al. [236] predicted the skin permeability of etodolac, famotidine, nimesulide, nizatidine, and ranitidine using a network trained with a dataset derived from the literature, and the predicted permeability agreed well with the experimental results. Tsuneji Nagai´s group successfully applied an ANN to optimize the amount of ethanol and O-ethylmenthol (causative factors) in a ketoprofen hydrogel formulation with respect to the response variables (penetration rate, lag time, and total irritation score (TIS)) in the late 1990s [238,239]. In addition, an ANN-based QSAR approach was applied to investigate the effect of 35 newly synthesized O-ethylmenthol derivatives on the percutaneous absorption of ketoprofen in vivo in male Wistar rats [204]. The calculated parameters (*logP*, *MW*, steric energy, van der Waals area, van der Waals volume, dipole moment, highest occupied molecular orbital, and lowest unoccupied molecular orbital) were used as derivative descriptors. Among them, *logP*, steric energy, and the lowest unoccupied molecular orbital significantly affected the prediction of enhancement factor (penetration rate with enhancer:penetration rate without enhancer) (Ef) and TIS (output variables). The predicted Ef and TIS values were in good agreement with the values obtained from in vivo percutaneous absorption experiments. In the study by Kandimalla et al. [205], the response surface method (RSM) and ANN were used to optimize the composition of the vehicle (water (W), ethanol (E), propylene glycol (P), and their binary and ternary mixtures) suitable to increase the flux and decrease the lag time of percutaneously applied melatonin. The prediction tools were a special quadratic model (RSM) and a back-propagation algorithm (ANN). RSM and ANN prediction of the best mixtures agreed very well. A non-linear QSPR model established by Yerramsetty et al. [206] used the ANN algorithm and an evolutionary algorithm (differential evolution (DE)) to predict the permeability enhancement of insulin when combined with 48 compounds with different functional groups as potential permeability enhancers. These compounds were identified using a virtual design algorithm, which combines genetic algorithms (GAs) and QSPR models to search for permeability enhancer descriptors. DE algorithms were applied to find the best descriptors and the best neural network architecture. In vitro permeation experiments using Franz diffusion cells were performed to quantify the effect of permeability enhancers. For ANN training, 35 compounds were used, while the remaining and additional 12 compounds reported in the literature were included in the validation set. The hybrid DE/ANN algorithm provided a good prediction for insulin permeability in the presence of the compounds studied. The statistically significant descriptors included: average 1-electron reactivity index for a carbon atom, minimum 1-electron reactivity index for an oxygen atom, Kier and Hall index (order 1), RNCS relatively negatively charged SA (SAMNEG*RNCG) [Zefirov’s PC], and total dipole of the molecule. The results obtained suggest that compounds with greater hydrophobicity and reactivity, as well as low dipole moments and the capacity to form intermolecular bonds with stratum corneum lipids, could be promising insulin-specific permeability enhancers.

The good predictive ability of the QSPR approach for single API solutions is well demonstrated. However, the QSPR model needs to be re-trained for formulations containing one or more excipients and does not provide insight into the mechanisms of permeation affected by chemical permeation enhancers. The MD simulations are a valuable addition to the QSPR methodology, allowing us to understand the location and orientation of excipients in the skin barrier structure at their most likely concentrations and how they interact with the barrier and influence the permeability of one or more APIs [224]. This can be a valuable tool for the selection of suitable excipients and the development of formulations tailored for a specific API. Lundborg et al. [224] proposed a new MD simulation using GROMACS 2021 and the second beta version of GROMACS 2022, which allows efficient sampling of the free energy and local diffusion coefficient to predict the skin permeability of 20 permeants with Mr between 18 and 300 gmol^−1^ and *logP* from 2.1 to 4.6. In addition, the penetration-enhancing effect of the chemical penetration enhancers DMSO, ethanol, and urea was analyzed. The simulations performed used the atomistic model of the human skin barrier structure originally called 33/33/33/75/5/0.3, corresponding to the relative composition in molar % ceramides/molar % cholesterol/molar % free fatty acids/relative amount of cholesterol on ceramide sphingoid side/molar % acyl ceramide EOS (included in the relative ceramide concentration)/water molecules per lipid (not included in the molar % concentrations of the lipids). The predicted properties of the permeating agents were in good agreement with the experimental in vitro skin permeation data (*logk_p_*). Such MD models enabled an understanding of the mechanisms of the effects of chemical penetration enhancers at the molecular level. For example, the permeability of codeine will be enhanced by a permeation enhancer acting in the ceramide fatty acid chain region rather than in the sphingoid chains. Iyer, with coworkers, combined classical and 4D fingerprint intramolecular QSAR descriptors to build two QSAR models [240,241]. The different mechanisms of skin permeation enhancement were proposed for datasets in which were varied the polarity and size of both the enhancer and the reference penetrant. The largest differences were between polar and nonpolar penetrants. Continued research focused on the development of membrane-interaction QSAR (MI-QSAR) models for two datasets of 61 and 42 penetrant enhancers to predict the improvement in skin permeability for hydrocortisone and hydrocortisone acetate [202]. The MI-QSAR model uses a model membrane (a phospholipid monolayer or bilayer) based on MD simulations, and a new MI-QSAR descriptor was developed, namely, the difference in the integrated cylindrical distribution functions over the phospholipid monolayer model, in and out of the presence of the skin penetration enhancer (ΔΣh(r)). The new descriptor was dominant in the optimized MI-QSAR models of both training sets. The MI-QSAR models were compared with QSAR models created for the same two datasets using only classical intramolecular QSAR descriptors. The new MI-QSAR descriptor reduces the size and complexity of the MI-QSAR models in comparison with classic QSAR models. The MI-QSAR models indicated that the skin penetration enhancer alters the structure and organization of the monolayer, which is constantly changing, so that the enhanced penetration is due to the creation of large or small “holes” in the monolayer. This alteration in the structure and dynamics of the membrane monolayer caused by each embedded skin penetration enhancer is captured by the new MI-QSAR descriptor. Stronger non-polar penetration enhancers cause larger “holes” in the monolayer. The MI-QSAR models explain 70–80% of the variance in skin penetration gain in each of the two training sets and are stable predictive models using recognized diagnostic measures of robustness and predictability.

## 6. Conclusions

In summary, the bio-performance of drugs, which includes release, dissolution, permeation, and absorption, is essential for their in vivo efficacy and bioavailability. In silico MIDD tools are increasingly important for understanding and optimizing these processes. While mechanistic models, often based on Fick’s law of diffusion, provide fundamental insights, their simplifications can lead to discrepancies between predictions and observations. Empirical models, on the other hand, are data-driven and form mathematical relationships based on experimental data using advanced techniques such as computational fluid dynamics. However, the complexity of drug release dynamics poses a challenge for both types of models. Recent advances in artificial intelligence, molecular dynamics simulations, and imaging techniques offer promising opportunities for more accurate modeling, particularly in percutaneous permeation studies.

Looking to the future, there is potential to integrate these advanced computational methods with evolving AI algorithms to improve predictive accuracy and interpretability. Further development in this area promises not only more efficient drug formulation and delivery but also a deeper understanding of the intricate interplay of factors that influence the bio-performance of drugs. This development underscores the importance of ongoing research and innovation in the field of in silico modeling for pharmaceutical development.

## Figures and Tables

**Table 1:** Examples of dissolution equations incorporated in PBB models.

Name	Equation	Denotations
Nernst–Brunner equation	$\frac{dM_{dissol}}{dt}=\frac{D\times A}{h}\times \left(C_{s}-C_{t}\right)$	*M_dissol_*—dissolved amount of drug *t*—time *C_s_*—solubility (saturation concentration) *C_t_*—drug concentration in solution at time t *D*—diffusion coefficient *h*—diffusion layer thickness *A*—effective surface area *ρ*—particle density *r*—spherical particle radius *s*—shape factor *L*—particle length *d*—particle diameter *M_undissol_*—undissolved amount of drug at time t *M_undissol(0)_*—initial amount of the solid drug *z* *—hybrid dissolution parameter
Johnson equation	$\frac{dM_{dissol}}{dt}=\frac{D}{h\times \rho \times r}\times \frac{1+2s}{s}\left(C_{s}-C_{t}\right)\times M_{undissol}$ $s=\frac{L}{d}$	
Wang–Flanagan equation	$\frac{dM_{dissol}}{dt}=\frac{3D}{\rho \times r}\times (\frac{1}{h}+\frac{1}{r})\times \left(C_{s}-C_{t}\right)\times M_{undissol}$	
*z*-parameter equation *	$\frac{dM_{dissol}}{dt}=z\times \left(C_{s}-C_{t}\right)\times M_{undissol\left(0\right)}\times {\left(\frac{M_{undissol}}{M_{undissol\left(0\right)}}\right)}^{2/3}$ $z=\frac{3D}{h\times \rho \times r}$	

* *z*-parameter can be used as a substitute for the effect of drug particle size, density, diffusion coefficient, and diffusion layer thickness on drug dissolution rate when these parameters are considered constant for a given formulation under the specific dissolution condition; *z*-parameter is formulation-specific [21].

**Table 2:** Overview of diffusion models for reservoir devices and matrix systems of different geometries.

Device Type	Slab	Sphere	Cylinder
Reservoir device with non-constant activity source $c_{ini}$ < $c_{s}$	$\frac{M_{t}}{M_{\infty }}=1-exp\left(-\frac{ADKt}{VL}\right)$	$\frac{M_{t}}{M_{\infty }}=1-exp\left(-\frac{3R_{o}DKt}{R_{i}^{2}R_{o}-R_{i}^{3}}\right)$	$\frac{M_{t}}{M_{\infty }}=1-exp\left(-\frac{\left(R_{i}H+R_{o}H+2R_{i}R_{o}\right)DKt}{R_{i}^{2}R_{o}-R_{i}^{3}}\right)$
Reservoir device with constant activity source $c_{ini}$ > $c_{s}$	$M_{t}=\frac{ADKc_{s}}{L}t$	$M_{t}=\frac{4\pi DKc_{s}R_{o}R_{i}}{R_{o}-R_{i}}t$	$M_{t}=\frac{2\pi HDKc_{s}}{\ln(R_{o}/R_{i})}t$
Matrix systems as monolithic solutions $c_{ini}$ < $c_{s}$	$\frac{M_{t}}{M_{\infty }}=1-\frac{8}{\pi^{2}}\overset{\infty }{\underset{n=0}{\sum }}\frac{exp\left[-D{\left(2n+1\right)}^{2}\pi^{2}t/L^{2}\right]}{{\left(2n+1\right)}^{2}}$	$\frac{M_{t}}{M_{\infty }}=1-\frac{6}{\pi^{2}}\overset{\infty }{\underset{n=1}{\sum }}\frac{exp\left[-Dn^{2}\pi^{2}t/R^{2}\right]}{n^{2}}$	$\frac{M_{t}}{M_{\infty }}=1-\frac{32}{\pi^{2}}\overset{\infty }{\underset{n=1}{\sum }}\frac{1}{q_{n}^{2}}\exp(-\frac{q_{n}^{2}}{R^{2}}Dt)\cdot \overset{\infty }{\underset{p=0}{\sum }}\frac{1}{{\left(2p+1\right)}^{2}}\cdot \exp(-\frac{{\left(2p+1\right)}^{2}\pi^{2}}{H^{2}}Dt)$
Matrix systems as monolithic dispersions $c_{ini}$ > $c_{s}$	$M_{t}=A\sqrt{Dc_{s}\left(2c_{ini}-c_{s}\right)t}$	$\frac{M_{t}}{M_{\infty }}-\frac{3}{2}\left[1-{\left(1-\frac{M_{t}}{M_{\infty }}\right)}^{\frac{2}{3}}\right]=\frac{3D}{R^{2}}\cdot \frac{c_{s}}{c_{ini}}\cdot t$	$\frac{M_{t}}{M_{\infty }}+\left(1-\frac{M_{t}}{M_{\infty }}\right)ln\left[1-\frac{M_{t}}{M_{\infty }}\right]=\frac{4D}{R^{2}}\cdot \frac{c_{s}}{c_{ini}}\cdot t$

where A—total surface area of the device, $c_{ini}$—initial concentration of the drug in the device, $c_{s}$—solubility of the drug, D—diffusion coefficient of the drug, H—length of the cylinder, K—partition coefficient of the drug between the membrane and the reservoir, L—thickness of the membrane, $M_{t}$—cumulative amount of the drug released at the time *t*, $M_{\infty }$—cumulative amount of the drug released at infinity, R—radius of the sphere, $R_{o}$—outer radius of the device, $R_{i}$—inner radius of the device, *t*—time, V—volume of the reservoir.

**Table 3:** Interpretation of Peppas equation exponent depending on the delivery system geometry.

Delivery System Geometry			Release Mechanism
Thin Film	Cylinder	Slab	
0.5 0.5 < n < 1.0	0.45 0.45 < n < 0.89	0.43 0.43 < n < 0.85	*Fickian* diffusion Anomalous transport (combined mechanisms)
1.0	0.89	0.85	*Case II* transport (usually synchronized swelling and erosion of polymers)
>1.0	>0.89	>0.85	*Super Case II* transport

**Table 4:** Selected examples of imaging studies used for analysis of the dissolution process.

Method	API, Delivery System/Dosage Form	Studied Process	References
ATR-FTIR * imaging	Ibuprofen (acid and salt formulations) in amorphous solid dispersions produced through hot-melt extrusion with copovidone and Soluplus^®^	Interaction of different forms of ibuprofen with polymers: in extrudates with its acidic form, ibuprofen was found to interact with polymers by forming hydrogen bonds, resulting in more sustained drug release.	[83]
	Ibuprofen (crystalline and amorphous form) in physical mixtures (PM) and hot-melt loaded (HML) mesoporous silica microparticles	Based on the chemical images, the faster release of amorphous ibuprofen from HML tablets compared to crystalline ibuprofen in PM tablets was observed. Ibuprofen dissolved from the PM tablets was adsorbed on the surface of the silica particles.	[84]
	Indomethacin formulated with nicotinamide, urea, and mannitol in different ratios	The observed changes in the release kinetics of indomethacin (from first-order to zero-order) can be interpreted from the results of the spatial distribution of the components during the dissolution.	[85]
UV-Vis imaging system	Placebo hydrophilic matrix tablets made of two HPMC ** grades	The swelling behavior of hydrophilic matrices of two HPMC grades with different particle morphology and using two compression forces.	[86]
	Metformin extended-release tablets	The release of metformin and the swelling of the polymer matrix were monitored simultaneously (at 255 nm and 520 nm, respectively).	[87]
	Propranolol formulated in liqui-solid compacts of Sesamum radiatum gum	Differences in the release behavior of propranolol from physical mixtures and liqui-solid formulations were observed.	[88]
UV-imaging system	Tablets with paracetamol or carbamazepine were formulated with super disintegrants (sodium starch glycolate or croscarmellose sodium)	The influence of the properties of the active substance and the properties and variability of the excipients on the release of the drug were investigated.	[89]
NIR ***-imaging system	Paracetamol in hydrophilic matrix tablets	Coupling hydrodynamic studies with NIR chemical imaging and dissolution data provided new insights into the mechanisms of drug release.	[90]

* ATR-FTIR—attenuated total reflectance-Fourier transformed infrared spectroscopy, ** HPMC—hydroxypropyl methylcellulose, *** NIR—near-infrared spectroscopy.

**Table 5:** Examples of permeability equations incorporated in PBB models.

Process	Equation	Denotations
Passive diffusion	$\frac{dM}{dt}=\frac{D\times A}{h}\times \left(C_{1}-C_{2}\right)$ (Fick´s first law of diffusion)	*dM*/*dt*—drug diffusion rate *D*—diffusion coefficient *A*—membrane surface area *h*—membrane thickness *C*_1_—concentration in the GI lumen *C*_2_—concentration in the blood *P*—partition coefficient between the lipid membrane and GI fluids *V*—uptake rate *V_max_*—maximum uptake rate *K_m_*—Michaelis–Menten constant *C_subs_*—substrate concentration *P_para_*—paracellular permeability *ε*—porosity *δ*—pore length *F*(*r*/*R*)—Renkin function *r*—drug molecular radius *R*—radius of the pore *κ*(*z*)—electrochemical energy function (for the charged species with *z* valence)
	$\frac{dM}{dt}=\frac{D\times A\times P}{h}\times \left(C_{1}-C_{2}\right)$ (Modified Fick´s first law of diffusion)	
Active transport	$V=\frac{V_{max}\times C_{subs}}{K_{m}+C_{subs}}$ (Michaelis-Menten equation)	
Convective (paracellular) transport	$P_{para}=\frac{\varepsilon \times D}{\delta }\times F\left(\frac{r}{R}\right)\times \frac{k\left(z\right)}{1-e^{k\left(z\right)}}$ (Adson equation)	

**Table 6:** Common methods for drug permeability determination.

Method	Equation	Denotations
Non-cell-based methods (e.g., PAMPA * test)	Papp=dQdtA×C0	*P_app_*—apparent permeability coefficient *P_eff_*—effective permeability *dQ*/*dt*—permeability rate *A*—membrane surface area *C*_0_—initial drug concentration *Q*—perfusion flow rate *C*_*in*′_—inlet drug concentrations adjusted for water transport *C*_*out*′_—outlet drug concentrations adjusted for water transport *R*—radius of the perfused intestinal segment *L*—length of the perfused intestinal segment
Cell-based methods (e.g., Caco-2 cells **, MDCK cells ***)		
Animal models (e.g., rat)	Peff=−Q×lnCout′Cin′2πRL	
Human studies (e.g., *Loc-I-Gut* [141])	$P_{eff}=\frac{Q\times \left(C_{in}-C_{out}\right)}{2\pi RLC_{out}}$	

* PAMPA—parallel artificial membrane permeability; ** Caco-2—human colorectal adenocarcinoma cell line; *** MDCK—Madin–Darby canine kidney cell line.
